# Implications of maternal-fetal health on perinatal stem cell banking

**DOI:** 10.1038/s41434-023-00426-w

**Published:** 2023-10-26

**Authors:** Dandan Zhu, Mehri Barabadi, Courtney McDonald, Gina Kusuma, Ishmael Miguel Inocencio, Rebecca Lim

**Affiliations:** 1 The Ritchie Centre, Hudson Institute of Medical Research, Clayton, VIC Australia; 2 Department of Obstetrics and Gynaecology, Monash University, Monash, VIC Australia

**Keywords:** Stem cells, Biotechnology

## Abstract

Cell based therapies are being assessed for their therapeutic potential across a variety of diseases. Gestational tissues are attractive sources for cell therapy. The large number of births worldwide ensures sufficient access to gestational tissues, however, limited information has been reported around the impact of birth trends, delivery methods and pregnancy conditions on perinatal stem cell banking. This review describes the current state of banking of gestational tissues and their derived perinatal stem cells, discusses why the changes in birth trends and delivery methods could affect gestational tissue banking practices, and further explores how common pregnancy complications can potentially influence perinatal stem cell banking.

## Introduction

Cell based therapies have been heralded as the latest pillar of modern medicine [1] and are currently being assessed for their suitability in treating a variety of diseases. Stem cells for therapeutic purposes can be isolated from a number of sources including induced pluripotent stem cells and embryonic, fetal, adult and gestational tissue. Gestational tissues comprise the placenta, including the amnion and chorion, and umbilical cord tissue and umbilical cord blood. Gestational tissues are an attractive source for stem and stem-like cells as they are rich in regenerative cell types without legal, ethical or moral concerns. We recognise that the regenerative cell types found within gestational tissues include both stem and stem-like cells. Throughout this review, we will refer to them collectively as ‘perinatal stem cells’. While there is a multitude of pre-clinical studies and clinical trials investigating the potential of perinatal stem cells, the impact of birth trends, delivery methods and pregnancy conditions on perinatal stem cells is limited. We will discuss changes in birth trends and explore how common maternal-fetal complications can potentially influence perinatal stem cell banking practices and stem cell quality attributes.

## Clinical use of perinatal stem cells

**Fig. 1:**
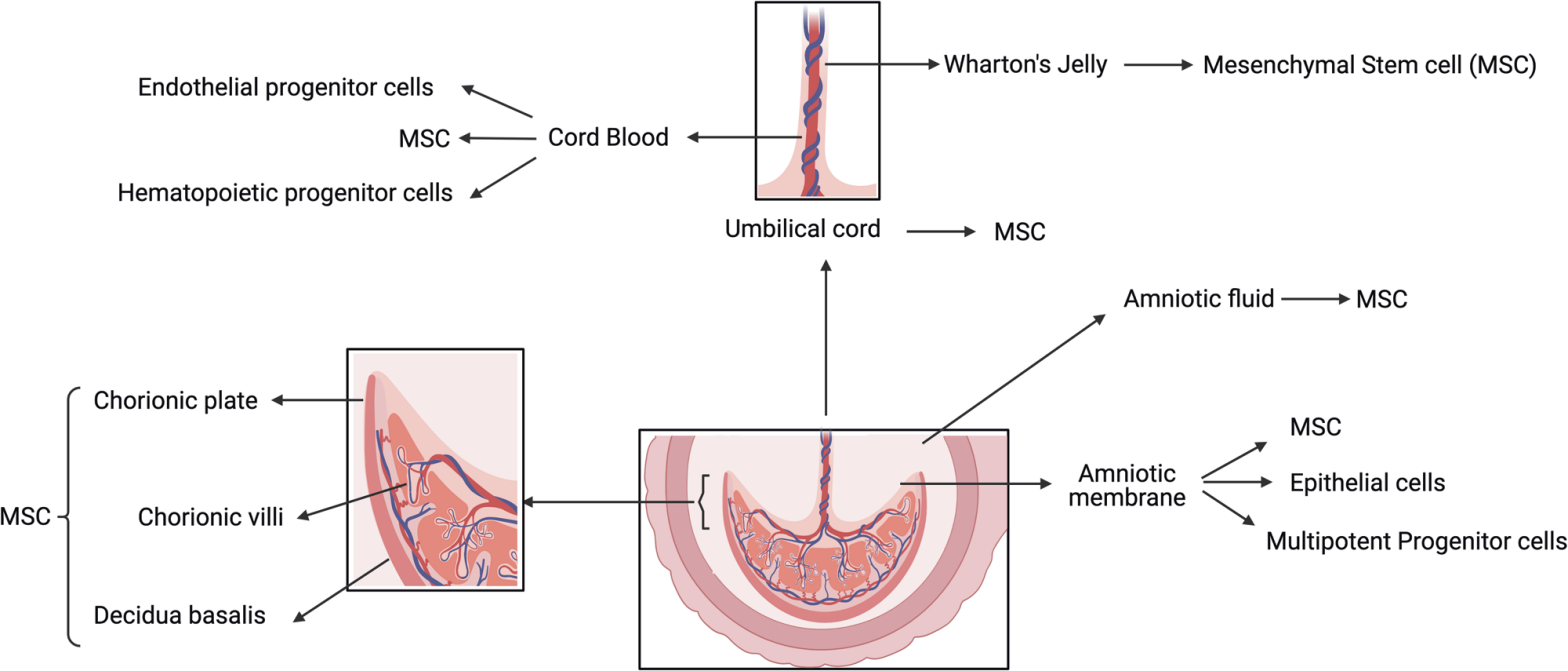
Schematic diagram of perinatal stem cells.

Gestational tissues give rise to stem cell types such as hematopoietic stem/progenitor cells (HSCs/HPCs), endothelial progenitor cells (EPCs), mesenchymal stem/stromal cells (MSCs) and human amnion epithelial cells (hAECs) (Fig. 1). HSCs/HPCs are an FDA approved cellular therapy product used clinically for a number of indications including bone marrow failure, haematological malignancies, congenital immunodeficiency syndromes, hemoglobinopathies and inherited metabolic diseases [2–5]. The use of HSCs for non-homologous applications, and other perinatal cell types are still under investigation.

**Fig. 2:**
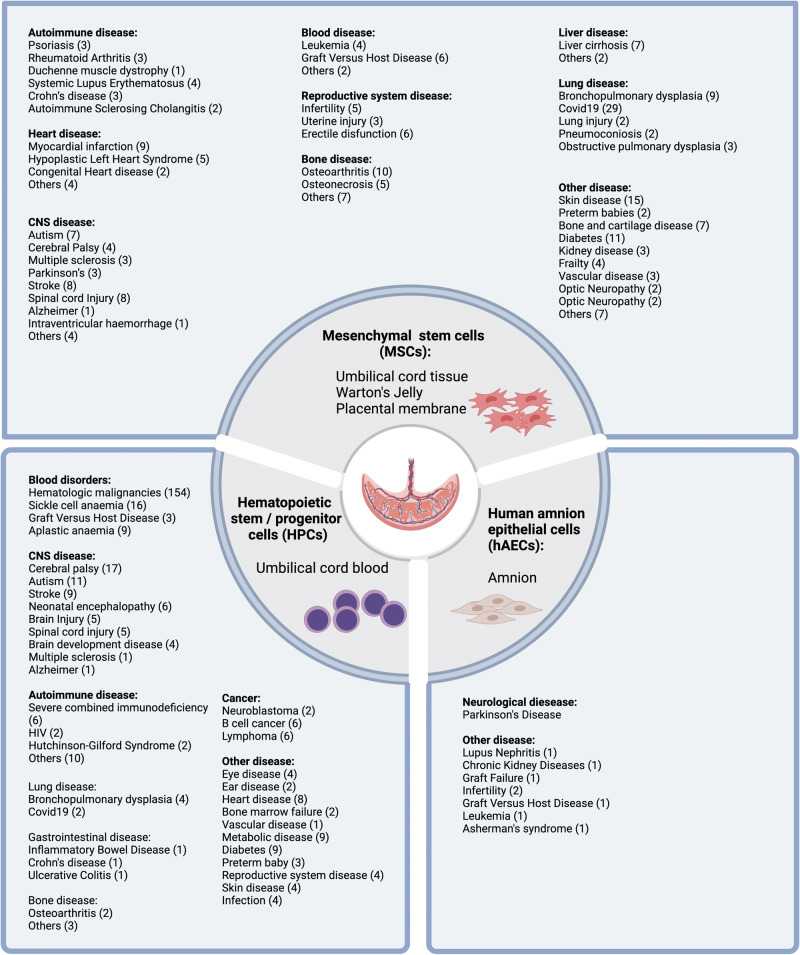
Clinical trials employing perinatal stem cells (data derived from https://www.clinicaltrials.gov).

Our appreciation of the potential of perinatal stem cells has improved significantly in the past decade. For example, cord blood is now understood to contain a mixture of stem cells including HSCs, EPCs and MSCs. HSCs are the most commonly studied stem cell population in cord blood, approved for treatment of some haematological, genetic and immunodeficiency diseases. Additionally, the therapeutic potential of cord blood has been investigated in disorders [6] such as hypoxic-ischemic encephalopathy [7], stroke [8], autism [9], and cerebral palsy [10]. Similarly, umbilical cord derived MSCs were reportedly therapeutic in disease settings such as rheumatoid arthritis [11] myocardial infarction [12], heart failure [13], and hypoxic ischemic encephalopathy [14]. Recently, fetal-liver-derived MSCs were used for in utero and postnatal treatment for osteogenesis imperfecta [15]. Compared to most other perinatal cell types, hAEC can be isolated in sufficient numbers for clinical use without the need for expansion [16]. hAECs are in clinical testing for diseases including stroke [17], bronchopulmonary dysplasia [18, 19], end-stage liver diseases (ACTRN 12616000437460) and Crohn’s disease-related perianal fistulas (ACTRN 12618001883202). Clinical trials employing the above cell types are summarised in Fig. 2 (data derived from https://www.clinicaltrials.gov).

## Impact of pregnancy health and mode of delivery on perinatal cell products

There are more than 2000 clinical trials in the United States alone using perinatal stem cells, however, there is a scarcity of information around the impact of birth trends and delivery methods on the quality attributes of perinatal stem cells. Furthermore, allogeneic perinatal stem cells are largely limited to donations from healthy pregnancies. In this section, we will explore the impact of birth trends, delivery methods, and maternal-fetal health complications on perinatal stem cell banking practices and their therapeutic potential.

### Current state of banking of gestational tissues and their derived perinatal stem cells

The first clinical use of perinatal stem cells was the HSC fraction of cord blood for the correction of Fanconi’s anaemia [20], and it remains the only FDA approved perinatal stem cell therapy. Consequently, only umbilical cord blood is collected and stored in not-for-profit, government-funded cell banks. In contrast, other gestational tissues and their derived stem cells are banked within clinical centers, research organizations and privately funded companies.

The world’s first public umbilical cord blood bank was established in 1993 at The New York Blood Centre after the first successful cord blood derived HSC transplantation [21]. In the last 3 decades, the number of cord blood banks has grown significantly. At the time of writing this review, there are approximately 450 public and private cord blood banks worldwide [22]. A major debate in banking gestational tissues is public banking or private banking, which leads to the discussion of using autologous versus allogeneic cells for clinical translation, where private banks are largely built around autologous use business models.

Professional societies including the American Academy of Pediatrics, the American Society for Blood and Marrow transplantation, the International Federation of Gynecology and Obstetrics, and the United Kingdom’s Royal College of Obstetricians and Gynecologists have indicated a preference of public cord blood banking to private banking [23, 24] as public cord blood banking is free of charge to the donors and can be accessed to anyone who needs it. In contrast, private cord blood banks have a low percentage of use (about 1 in 1000) [25], are expensive to access (~2000 USD for collection and ~150 USD annual storage fee) [26], and provide limited family-only access. Consequently, private cord blood banking has not been recommended to families without a current or potential need for stem cell transplantation. Indeed, cord blood banking is only suggested when a family member has been identified as having a disease that is amenable to cord blood transplantation [27]. Nevertheless, more cord blood units are stored in private cord blood banks compared to public banks. By 2017, there were approximately 800,000 cord blood units stored in public cord blood banks [24], and more than 5 million cord blood units in private cord blood banks. This could be considered a waste of cord blood units that are banked privately at significant cost, with slim chances of being used. In contrast, 30-fold more cord blood units have been released for clinical purposes from public cord blood banks [25].

Given the significantly higher use of cord blood units from public banks, one can infer that there are also significantly more allogeneic cord blood transplantation cases compared to autologous. While the efficacy of autologous and allogeneic cord blood has not been compared within a single clinical study, cord blood from both sources have been found to improve the outcomes in cerebral palsy patients [10, 28–30]. In contrast, allogeneic cord blood but not autologous, has been shown to have therapeutic effect in Type I diabetes [31–33]. Thus, there is currently an absence of evidence to suggest that autologous cord blood transplantation achieves superior clinical outcomes.

There are increasingly more cord blood banks using hybrid models that combine aspects of both the public and private banking systems. There are two common hybrid models – one offers parents both public and private donation options, while the other makes privately stored cord blood available to the public [34]. Although there are concerns around technologies, standards, logistics, regulations and corporate ethics, these hybrid models address the gap between private and public banking [34, 35]. The emergent private-public banking model adds more privately banked cord blood units to the public pool, which increases the chance of being used either by patients or researchers [36]. It also improves transplantation opportunities for private patients when pooled cord blood is needed from multiple donors. In addition, it provides private banks with additional technical support from public banks and eases the pressure of staffing shortages [36].

The rate of cord blood banking varies across globally. Among the 54 countries that were taken into account by Cell Trials, the rate of cord blood banking ranged from 0.3% in UK to 30% in Singapore, with 27 countries reporting rates under 1% (https://celltrials.org). NetCord and the Foundation for the Accreditation of Cellular Therapy (NetCord-FACT) International Standards for cord blood collection, banking, and release for administration [37] suggests that donor criteria exclude genetic diseases, malignant diseases, inherited disorders, communicable diseases and human transmissible spongiform encephalopathy. However, there are no donor criteria around common pregnancy disorders and risk factors such as gestational diabetes, hypertensive disorders, preterm labour, smoking during pregnancy, and overweight or obesity. Whether gestational tissues from these common pregnancy disorders can be used as sources for perinatal stem cells depends on whether the potency of perinatal stem cells from these complicated pregnancies are affected in disease status. These are elaborated in detail in “The impact of pregnancy complications on perinatal cell banking”.

The NetCord-FACT criteria for cord blood units stored for allogeneic clinical use include the total nucleated cell (TNC) count ≥ 5.0 × 10^8^ with ≥ 60% recovery rate and ≥ 85% viability, viable CD34^+^ count ≥ 1.25 × 10^6^ with post-thaw viability ≥ 70%, and colony forming units [37]. Notably, these requirements vary between countries and banks. For instance, the requirements for cord blood banking in Japan is expected to rise to TNC of 10 × 10^8^ per cord blood unit, because of the anticipated increased need in adult populations due to haematological malignancies in their aging population [38]. An Italian cord blood bank has adopted a cut-off value of 8 × 10^8^ nucleated cells and a volume ≥60  mL [39]. A Mexican public cord bank sets cut-off values of ≥80 mL volume and ≥8 × 10^8^ TNCs [40]. Overall, the relevance of the cord blood volume and TNC numbers is dependent on the recipient and clinical indication. One can appreciate that the cord blood volume and TNC needed for haematological transplantation in a 5-year-old child will be different to that needed for an adult. Notably, partially HLA-matched cord blood units can be combined to overcome cell-dose limitations [41]. Furthermore, ex vivo expansion methods have been evaluated in an effort to achieve higher cell numbers from single-cord blood units. These include the use of HSC expansion cytokine cocktails comprising of granulocyte colony-stimulating factor (G-CSF), thrombopoietin (TPO), and Flt-3 ligand (Flt-3L) [42], reagents such as copper chelators [43] and nicotinamide [44], and bioreactor culture systems [45, 46]. While 2- to 400- fold expansion were reported pre-clinically and clinically [47], more clinical studies are needed to truly determine the feasibility and reproducibility of cord blood expansion.

Numerous cord blood banks also offer perinatal tissue banking, which includes umbilical cord tissue, placental tissue, and amniotic membrane. These tissues can be used for the derivation of EPCs, hAECs and MSCs. Concomitant collection of cord blood and birth tissues may be an economic approach for perinatal stem cell banking, where only a single donor would be screened for all the products [48]. NetCord-FACT standards of cord tissue collection and storage apply only to tissue samples collected for testing or research purposes, and FACT common standards for cellular therapies apply to birth tissues collected and stored for therapeutic intent [37]. Notably, there are no common criteria for donor selection, cell isolation and expansion from gestational tissues, or the clinical use of stored gestational tissues and their derived cells. Moreover, cell transplantations using hAECs and MSCs are usually for allogeneic use, and sometimes pooled cells from multiple donors are needed for sufficient doses.

### The impact of birth trends on perinatal stem cell banking

While global births have been stable in the last 30 years with a total of 138 million in 1980 and 141 million in 2015 (https://worldpopulationreview.com), maternal age has risen. In Australia, the median age of all mothers has increased from 26.3 years for all births registered in 1988, to 31.4 years in 2018 (https://www.abs.gov.au). The fertility rate of women aged 35–39 more than doubled in 2017, and for women aged 40–44, it tripled in the past 30 years in Australia. This has resulted in the birthing rates of mothers aged 35 years and over, increasing from 10% in 1978 to 24% in 2018 (https://www.abs.gov.au). In the USA, the average age of mothers was 28.8 years old in 2017. A similar trend in birth rates was observed with 5.3 births per 1000 mothers occurring in the 35–39 age group and 0.7/1000 in the 40–44 age group in 1987, which has increased by more than 10-fold to 52.3 and 11.6, respectively in 2017 [49].

To date, there have been limited reports on the impact of advanced maternal age on the functional activity of perinatal stem cells indicating a current gap that needs to be addressed. A recent study on placenta-derived mesenchymal stem cells from women aged >35 years showed lower self-renewal properties and proliferative capacity and lower expression of pluripotency and multipotency markers compared to women aged <35 [50]. Another recent study on Wharton’s Jelly-derived stem cells also showed significantly lower SOX2 gene expression in mothers aged >34 years indicating a negative correlation between maternal age and SOX2 gene expression [51]. SOX2 gene expression indicates stemness, proliferative and adhesion properties and cell migration in Wharton’s Jelly-derived stem cells. Similarly, another study reported a negative correlation between maternal age and gene expression of umbilical cord derived-MSC markers CD105 and CD29 [52].

Studies on the impact of advanced maternal age on the yield of perinatal stem cells are inconclusive. Some studies report no effect on TNC or CD34^+^ mononucleated cells in the cord blood units collected from mothers aged 35–40 years compared to mother aged 25–35 years [53]. While others report TNC of cord blood units from mothers aged over 30 years to be either similar or higher compared to younger mothers [38]. These results are aligned with another study reporting increased TNC in cord blood units from mothers aged between 20 to 37 years compared to women aged <20 and >37 years of age. This study also showed a negative correlation between the concentration of hematopoietic stem cells, regulatory T-cells (CD45^+^/CD4^+^/CD3^+^) and all lymphocytes (CD45^+^) in umbilical cord blood cell population and maternal age [54]. It is noteworthy, however, that advanced maternal age over 40 years, is an independent risk factor for preterm delivery, pregnancy-related hypertension disorders, gestational diabetes and abnormal fetal presentation [55, 56]. Further, advanced maternal age is associated with an increased risk of stillbirth, fetal growth restriction, and neonatal death [57, 58]. The potential impact of pregnancy disorders and risk factors on perinatal stem cells will be discussed later in this review “The impact of pregnancy complications on perinatal cell banking”.

### The impact of delivery methods on perinatal cell banking

Another consideration is the limitation placed on perinatal stem cell banking due to the method of delivery. Currently, cord blood can be collected from both vaginal and caesarean births. However, public donations of other gestational tissues usually require sterile collection from caesarean sections in an effort to avoid microbial contamination. Furthermore, majority of births including those that happen in hospitals are vaginal deliveries. In the USA, 68% of hospital births in 2017 were vaginal deliveries with 26% being low-risk cases [49]. In the same year, the rate of vaginal deliveries in Australia was similar, at ~70% (https://www.aihw.gov.au). Considering that most low-risk pregnancies are delivered vaginally, and majority of the research to date has focused on perinatal stem cells from healthy pregnancies, this severely limits the availability of potential donors for gestational tissues other than cord blood. While private banks offering tissue collection alongside with cord blood collection claim to bank tissues from both vaginal and caesarean births, there are concerns about sterility and perinatal stem cell quality when standardised collection procedures for vaginal births are lacking. At the time of writing, there are no published guidelines on bioburden reduction or indicating the time between collection and processing for tissues banked from vaginal births. Standard collection procedures should be developed in order to increase researcher access to tissues from low-risk/healthy pregnancies and standardise public and private banking procedures.

A further consideration with regards to the impact of delivery, specifically on cord blood collection, is the increasing trend of delayed cord clamping, where a delay of 60 s has been reported to significantly reduce blood volume collected and cell count [59]. Gestational tissues from home births are less likely to impact perinatal stem cell banking due to the low rates of home births. Home births remains below 5% in most countries despite a recent increase with 1.6% in US [60], 4% in New Zealand [61] in 2017, and 0.48% in Australia between 2000 and 2015 [62]. Home births are more common in countries like The Netherlands, accounting for approximating 20% of all births [63]. However, there are clear challenges in the sterile collection of gestational tissues in home birth settings, and timely processing for cell isolation.

### The impact of pregnancy complications on perinatal cell banking

Given that limited research has been undertaken to understand the impact of pregnancy complications and maternal health on perinatal stem cells, it is important to consider the significant proportion of caesarean deliveries that are affected by complications of maternal-fetal health.

#### Preterm birth

Preterm birth is defined as a live birth occurring before 37 weeks of pregnancy and is a common pregnancy complication, ranging from 5% to 18% of all the births in 2018 [64]. In 2016, preterm births accounted for 9.93% of all births in the US [49]. The most obvious impact of preterm birth on gestational tissue-derived stem cells is less starting material for cell isolation. Studies have shown that the volume of umbilical cord blood increases with gestational age where 21–62 mL of cord blood can be obtained from infants born at 22–33 weeks of gestational age and 49–90 mL from infants born at 34–37 weeks, compared to an average of 102 mL from term infants [65, 66]. Notably, TNC numbers also increased with gestational age where 2-3 × 10^8^ TNC could be collected from infants born at 22–33 weeks, 5–7 × 10^8^ TNC from infants born at 34–37 weeks, compared to an average of 11 × 10^8^ TNC from term infants [65, 66].

CD34 is a marker for haematopoietic progenitor cells in cord blood. The number of CD34^+^ cells in a cord blood unit is associated with engraftment, and it is superior indicator compared to TNC numbers for predicting engraftment [67, 68]. Notably, the concentration of CD34^+^ cells was found to be higher in cord blood collected from preterm infants, where the total number of CD34^+^ cells in cord blood units were similar in those collected from infants born between 22 and 36 weeks and those collected from term-born infants [66]. Others found that total CD34^+^ counts were highest in infants born at 34–37 weeks of gestation compared to those who were born at 28–33 weeks and 38–41 weeks [65]. The proliferative and self-renewal capacity of CD34^+^ cells in cord blood collected from preterm infants have been shown to be higher than that in term-born infants [69]. Similarly, endothelial colony-forming cells (ECFC) from preterm cord blood were greater in number and had higher proliferation rates compared to ECFCs from term cord blood [70]. In contrast, MSCs derived from term and preterm umbilical cords had similar proliferation rate and colony-forming unit efficiency [71]. Nevertheless, there are controversies on the potency of perinatal stem cells from preterm birth. While conditioned media from preterm umbilical cord-derived MSCs has been reported to ameliorate alveolar simplification and pulmonary inflammation in hyperoxia-induced lung injury, prematurity has also been reported to negatively impact regenerative properties of perinatal stem cells [72]. In an ovine model of preterm perinatal brain injury, both preterm and term cord blood cells were able to reduce cell death, white matter injury and inflammation, while term cord blood cells were more effective at reducing oxidative stress than their preterm counterparts [73]. A similar study showed that hAECs isolated from preterm gestational tissues were shown to be less effective in lung repair [74].

As discussed earlier, umbilical cord blood from preterm birth could be banked for hematopoietic disorders such as leukemia, anaemia and autoimmune disorders. Indeed, there have been some feasibility clinical studies using autologous preterm cord blood transfusion for anaemia with contradicting conclusions. Some claimed autologous cord blood as an effective therapeutic with limited side effects [75] while others suggest that autologous cord blood from preterm infants cannot replace 60–70% of allogeneic transfusions due to the low volume [76, 77]. Selective preterm cord blood banking may be promising, but prospective collection and cord blood banking technology must be improved, and clinical efficacy should be confirmed through larger-scale clinical trials.

#### Hypertension disorders during pregnancy

Hypertension disorders during pregnancy (HDP) accounts for 3–16% maternal mortality [78, 79] and precedes 7% of early neonatal deaths [78]. The histological abnormalities in placentas from HDP include reduced vascularity, greater placental infarction, villous fibrinoid necrosis, and villous hypermaturity [80]. These findings indicate placental ischemia, leading to oxidative stress and chronic-fetal hypoxemia. HDP includes chronic hypertension, gestational hypertension, preeclampsia/eclampsia, and preeclampsia superimposed on chronic hypertension [81]. Preeclampsia affects 6% of all deliveries according to a global survey representing 39 million women from 40 countries [82]. It is one of the main causes for maternal, fetal and neonatal mortality and the only effective treatment is delivery of the fetus and placenta [83].

The number of EPCs isolated from umbilical cord blood was reportedly lower in preeclamptic patients compared to healthy patients, and the proliferation, migration and vasculogenic capacities of EPCs were impaired by preeclampsia [84]. However, others have shown that EPC numbers from maternal peripheral blood did not change and EPC proliferation was actually higher in preeclamptic women [85]. Studies have also shown that preeclampsia can affect other perinatal stem cells. Blood volume, number of TNCs and colony forming units were reduced in preeclamptic umbilical cord blood compared to healthy term cord blood, however, the expression of cell adhesion molecules such as lymphocyte function-associated antigen-1(LFA-1), very late activin antigen-4 (VLA-4) and L-selectin were unchanged. Also, placental decidua-derived MSCs, but not amniotic membrane-derived MSCs, from preeclamptic placentae had reportedly lower levels of soluble intracellular adhesion molecule-1 (sICAM-1) and stromal-derived factor-1 (SDF-1) [86] which may explain a reduction in MSC migration to sites of injury. Decidua basalis-derived MSCs isolated from preeclamptic placentae expressed significantly lower aldehyde dehydrogenase enzyme activity which is associated with its ability to respond to oxidative damage, a hallmark of preeclampsia [87]. There are yet no in vivo studies to further assess the therapeutic effect of perinatal stem cells from preeclamptic pregnancies.

#### Gestational diabetes

The global median estimates of gestational diabetes mellitus (GDM) range from 6% to 13% [88]. While there are limited studies on the impact of gestational diabetes on perinatal stem cells, one study reported reduced numbers of circulating EPCs in cord blood from GDM affected pregnancies [89]. Umbilical cord derived MSCs from GDM affected pregnancies have reportedly low proliferative rates, reduced cell viability, increased cell death, and low mitochondrial activity [90, 91]. Furthermore, placental MSCs from GDM-affected pregnancies were found to be insulin-resistant and exhibited decreased clonogenicity and angiogenic potential [92]. Chorionic MSCs from GDM-affected pregnancies had increased adipogenic potential but similar ability to suppress T cell proliferation compared to MSCs from healthy pregnancies [93]. These limited studies indicate that GDM may influence subpopulations of perinatal stem cells differently and it is yet unknown how disease management can influence cell yield and quality. The impact of insulin or metformin on one of more stem cells may be different to dietary modification. Knowledge in this area will be critical in developing donor criteria for perinatal stem cell banking.

#### Smoking during pregnancy

While the global prevalence of smoking is generally low, 1 in 14 women (7.2%) birthed in the US in 2016 reported smoking during pregnancy [94]. Women who smoked for more than 3 months before pregnancy accounts for 9% of all pregnancies in US in 2017 [49]. Prevalence of smoking was highest among women aged 20–24 (10.7%), followed by women aged 15–19 (8.5%) and 25–29 (8.2%) [94]. In addition to the known effects of maternal smoking on fetal health, one study showed that maternal smoking correlated with lower mononuclear cell viability and increased oxidative stress proteins products in the umbilical cord blood [95]. However, there has been no study to date describing the effects of maternal smoking on the yield or potency of perinatal stem cells.

#### Intrauterine growth restriction

Intrauterine growth restriction (IUGR) is a complex maternal, fetal and placental-related condition leading to an increased risk of perinatal mortality and morbidity. Infants born with IUGR have an estimated weight less than the 10th percentile caused by insufficient transfer of oxygen, nutrients and metabolites in the uterus [96]. While the prevalence of IUGR is between 5–15% in US and Europe, developing countries report a wide range of 10 to 55% prevalence [97]. A comparison between the amniotic membrane-derived MSCs isolated from IUGR compared to healthy placenta indicates a lower level of cell proliferation, angiogenesis capacity and restricted multipotency. While IUGR derived MSCs showed a higher differentiation capacity to adipose tissue in vitro, their capacity to differentiate toward endothelial cell lineage was reduced [98]. Cord blood-derived EPCs from IUGR pregnancies have also been shown to be fewer in number and less vasculogenic compared to healthy pregnancies, due to over expressing genes such as thrombospondin-1 [99, 100]. Comparison of stem cells isolated from healthy term pregnancies against IUGR pregnancies may be challenging due to the differences in gestational ages, however this is an area where further research is warranted.

Apart from the major pregnancy disorders that have been mentioned above, there are other conditions that may affect the banking of perinatal stem cells. For example, the incidence of pregnancies resulting from infertility treatment has grown in recent years. It accounts for 1.87% of total births and approximately 20% of all multiple births in US [101]. In UK, 12.5% women were reportedly infertile [102], and 4.2% of women aged 40–55 reported that they had achieved at least one pregnancy from infertility treatment [103]. There has been over 8 million babies born from in vitro fertilization (IVF) treatment worldwide [104], and the majority of infertility treatment usually requires the use of drugs and medical procedures. It is unclear if either or both impact the therapeutic potential of perinatal stem cells. Moreover, studies showed that there was higher risk of adverse pregnancy outcomes from infertility treatment, such as GDM, HDP, very low birthweight, very preterm birth and neonatal death [105, 106].

The use of medications for pre-existing diseases during pregnancy should also be considered. For example, women with systemic lupus erythematosus (SLE), asthma, and hypothyroidism are advised to continue medications including hydroxychloroquine, corticosteroid, or thyroxine during pregnancy. While no significant increase in adverse pregnancy outcomes has been identified [107–109], it is worth noting that the impact of chronic diseases and long-term medication on perinatal stem cells has not been investigated.

## Conclusion

Gestational tissues are attractive sources for stem cells. The large number of births that occur globally ensures sufficient access to gestational tissues, however, the increasing trend in maternal age demands future research on the impact of geriatric pregnancies on perinatal stem cell yield and quality. Furthermore, standard collection procedures should be developed for gestational tissues from vaginal birth, beyond umbilical cord blood. This would enable the collection of gestational tissues from majority of births, rather than limiting collections to caesarean sections. Importantly, further research on the functional activity and therapeutic potential of perinatal stem cell derived from unhealthy pregnancies or women with advanced maternal age is needed to investigate the impact of perinatal stem cell banking practices on the efficacy of cell therapies. These studies should include experimental testing of the perinatal stem cells in disease models, and potentially, large-scale clinical studies to provide valuable information for development of donor criteria with regards to perinatal stem cell banking.

## Data Availability

All data as part of this study are included in this published article.
